# Uses for humanised mouse models in precision medicine for neurodegenerative disease

**DOI:** 10.1007/s00335-019-09807-2

**Published:** 2019-06-15

**Authors:** Remya R. Nair, Silvia Corrochano, Samanta Gasco, Charlotte Tibbit, David Thompson, Cheryl Maduro, Zeinab Ali, Pietro Fratta, Abraham Acevedo Arozena, Thomas J. Cunningham, Elizabeth M. C. Fisher

**Affiliations:** 1 Mammalian Genetics Unit, MRC Harwell Institute, Oxfordshire, OX11 0RD UK; 2 Unidad de Investigación Hospital Universitario de Canarias, FUNCANIS, Instituto de Tecnologías Biomédicas ULL, and CIBERNED, La Laguna, 38320 Tenerife, Spain; 3 Department of Neuromuscular Diseases, Institute of Neurology, University College London, London, WC1N 3BG UK

## Abstract

Neurodegenerative disease encompasses a wide range of disorders afflicting the central and peripheral nervous systems and is a major unmet biomedical need of our time. There are very limited treatments, and no cures, for most of these diseases, including Alzheimer’s Disease, Parkinson's Disease, Huntington Disease, and Motor Neuron Diseases. Mouse and other animal models provide hope by analysing them to understand pathogenic mechanisms, to identify drug targets, and to develop gene therapies and stem cell therapies. However, despite many decades of research, virtually no new treatments have reached the clinic. Increasingly, it is apparent that human heterogeneity within clinically defined neurodegenerative disorders, and between patients with the same genetic mutations, significantly impacts disease presentation and, potentially, therapeutic efficacy. Therefore, stratifying patients according to genetics, lifestyle, disease presentation, ethnicity, and other parameters may hold the key to bringing effective therapies from the bench to the clinic. Here, we discuss genetic and cellular humanised mouse models, and how they help in defining the genetic and environmental parameters associated with neurodegenerative disease, and so help in developing effective precision medicine strategies for future healthcare.

## Introduction

Neurodegenerative diseases are characterised by progressive loss of neuronal subsets in the brain or spinal cord and afflict millions worldwide. These disorders are a leading cause of premature death globally, and have devastating social and personal costs to those affected and those who are close to them (Livingston et al. 2017). Dementia—the dysfunction/loss in multiple cognitive areas that can arise from neurodegeneration—has reached pandemic proportions, afflicting 50 million people in 2018, with the annual economic cost currently estimated at $1 trillion (Patterson 2018). Cases are on course to triple by 2050 largely due to our ageing population, with dramatically increased prevalence in those over 65 years of age (Patterson 2018).

Neurodegeneration is not a disease of the relatively affluent—dementia incidence is plateauing in high-income countries, but increasing in low- and middle-income countries, which have poorer access to healthcare and education (Patterson 2018). Neurodegeneration is also not necessarily a disorder of ageing: a minority of neurodegenerative disease affects even the very young; for example, the single biggest genetic killer of infants remains type 1 spinal muscular atrophy (SMA) with an incidence of up to 1 in 6000 newborns in different populations worldwide (Sugarman et al. 2012). Even typically late-onset diseases can occur, exceptionally, in young people. Amyotrophic lateral sclerosis (ALS) has been described in 11-year old children; Alzheimer's disease (AD) typically occurs in the 4th decade of life in people who have Down syndrome (DS); and Parkinson’s disease (PD) can manifest before the age of 30 (Conte et al. 2012; Trinh et al. 2019; Wiseman et al. 2015).

Currently, we have at best very limited therapeutic options for most neurodegenerative disorders, and cures for none of them. However, this bleak outlook may be changing slowly and there is hope on the far horizon for at least some patients, with the advent of new therapies such as the use of antisense oligomers, gene therapies, antibody therapies, and stem cell therapeutics, in addition to conventional small molecules.

However, human and mouse phenotypes result from a largely undefined mix of genetics, environment, ageing, and stochastic effects, all of which need to be investigated to determine how to best treat and prevent disease. Neurodegeneration has a heterogeneous manifestation, so even in cases where known genetic mutation is causal for disease, we still only understand a small part of the whole picture of pathogenesis. For example, a triplet repeat expansion in the Huntingtin gene causes ~ 100% Huntington disease (HD), and while the size of this expansion largely determines age of disease onset, other alleles in the genetic background also modulate this timepoint (Long et al. 2018).

Neurodegenerative diseases, even the genetic forms, have complex etiologies, so they will require stratified treatments. Therefore, to create these treatments, we need real world data from human populations, which is usually highly variable and noisy. We also need models we can manipulate, to help us tease out mechanistic insight into disease, and to assess therapies. This is particularly challenging for the late-onset disorders in which disease typically manifests after many decades with no presymptomatic indications that we know of.

Different model systems provide different information and insights, but all are important for building the picture of pathomechanism. This includes in vitro systems, such as using human neurons produced from human pluripotent stem cells (PSCs, derived from embryos) or induced pluripotent stem cells (iPSCs), and in vivo systems, such as animal models of human disease. These animals may model genetic forms of neurodegeneration, or have alleles that modulate disease manifestation, or be exposed to varied environments to assess effects on disease outcome (Hockly et al. 2002).

All research results from model systems must be validated in the human population, which is considerably more variable than any laboratory system. The long-term goals are to find treatments and ultimately cures that work in different individuals and also, critically, to use these in real world healthcare systems where cost-effectiveness and realistic medical regimes are the only options.

### Precision medicine: contributions from the mouse

**Fig. 1:**
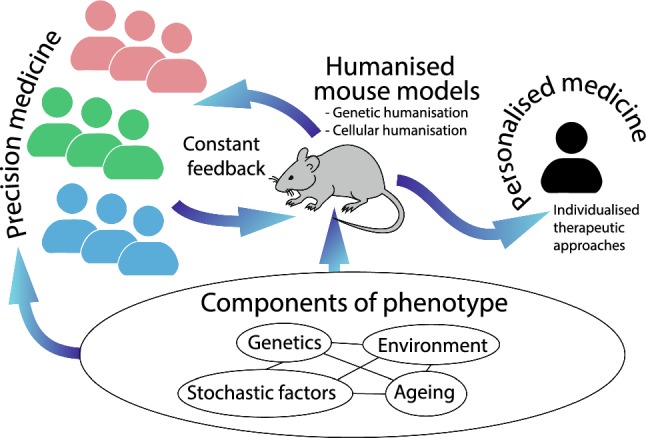
An illustration of the role humanised mouse models play in the development of new precision and personalised medicine strategies

Precision medicine refers to an evidence-based approach to healthcare, in which the best treatment for an individual is chosen based on their genetic/epigenetic make up and other features such as their microbiome, age, nutrition, lifestyle, and specific biomarkers. Clearly, the more we understand, the more we can stratify treatments into the correct patient sub-groups, not just by genetics but by all other factors that make us distinctive individuals (Fig. 1).

To create precision medicine strategies requires a systems-based approach (Berlin et al. 2017), very large datasets, new algorithms, machine learning, many different types of data input, combined with rigorous statistical analysis to determine what is optimal in different sub-groups—and this can include even lifestyle changes such as in diet or exercise. The size of the problem means that large collaborative networks of different clinical, translational, basic science, and computational specialists are required to contribute data and to analyse different outcomes; for review see (Strafella et al. 2018). There also remains an important role for the laboratory researchers drilling into pathomechanism, including those working with mouse models.

This review focuses on a specific type of mouse model, which is increasingly used in research: the humanised mouse. We discuss two types of humanised mouse—genetic and cellular humanised models and their different contributions to precision medicine for neurodegeneration.

### Mice are not human but we can humanise them

Despite many similarities, mice are obviously different from humans at all levels. Size, anatomy, neuroanatomy, lifespan, heart rate, reproductive capacity, and responses to drugs are clearly species specific and affect disease manifestation. Even at the genetic level, we do not share exactly the same set of genes: roughly 1% of mouse genes are not present in humans and vice versa (Mouse Genome Sequencing Consortium et al. 2002). Plus, humans have slightly more splice isoforms on average per gene (3.4 isoforms per protein coding gene) than mice (2.4) (Lee and Rio 2015). Nevertheless, we are separated by only 75 million years of evolution and biochemical mechanisms are broadly conserved between our species, and so mouse models have been a fruitful resource for understanding human disease [noting that more than 20 Nobel Prizes have been awarded for research primarily using mice (Festing and Fisher 2000)].

However, one way of potentially improving mouse models for studying human disease and neurodegeneration is to make the mice more ‘human’ either in terms of gene content by incorporating human DNA into the mouse genome (genetic humanisation) and/or cellular make up by engrafting human cells into mouse tissues (cellular humanisation). Remarkably, both approaches were pioneered over 30 years ago (Brundin et al. 1986; Gordon and Ruddle 1981; Gumpel et al. 1987; Stromberg et al. 1986; Wagner et al. 1981).

#### Genetic humanisation: incorporation of human DNA into the mouse genome

The reasons for humanising at the DNA level are so that mouse models express genes and proteins analogous to those found in people—perhaps with the human splice isoforms or with the human protein biochemistry that may be subtly but critically different from mouse protein biochemistry. For example, tauopathies are characterised by deposition of tau protein, encoded by the *MAPT* (microtubule-associated protein tau) gene, and these include frontotemporal dementia (FTD), corticobasal degeneration, progressive supranuclear palsy, Pick’s disease, and other rarer neurodegenerative disorders (Goedert et al. 2017). Humans express six tau isoforms in adult brain via alternative splicing, whereas mice express different isoforms in a different ratio—and this is critical for mouse modelling because human monogenic tauopathies can arise from *MAPT* mutations that affect the primary protein sequence and/or the splice isoform ratios (Goedert et al. 2017). Therefore, human *MAPT* sequences are required to model human tauopathies and so almost all mouse models of tau deposition are transgenic animals expressing the human gene.

Genetic humanisation can be achieved through a number of different strategies (Table 1). Injection of plasmids or artificial chromosome vectors into mouse zygotes results in the random genomic integration of human transgenes. This approach was used to generate the earliest mice created with human transgenes (Gordon and Ruddle 1981; Wagner et al. 1981), and is still frequently used to introduce human transgenes harbouring pathogenic mutations. Transgenic models have been the bedrock of neurodegeneration research, and have contributed important results for understanding human disease. However, they have some key features which we must consider, including that transgenes concatermerise when they insert into the genome, which they do randomly. Therefore, most transgenics, whether made from cDNA or genomic DNA, in plasmids or larger constructs, tend to be in multiple copies and thus overexpress the protein of interest. Furthermore, several transgenic models utilise a non-endogenous promoter, such as the prion promoter, that can also lead to increased expression over endogenous levels. Overexpression can be helpful in that it may increase the rate of phenotype progression, as with the most widely used ALS mouse model, the SOD1-G93A humanised transgenic mouse (Gurney et al. 1994). ‘High-copy’ mice reach humane endpoint on commonly used genetic backgrounds before ~ 140 days, whereas ‘low-copy’ animals, which are identical apart from having deleted much of the transgene array, take up to 9 months to reach humane endpoint (Acevedo-Arozena et al. 2011). Moreover, in the majority of transgenic models, the human sequence is expressed together with the mouse endogenous gene. Many genes/proteins, including the ALS-associated RNA-binding proteins, are highly dosage-sensitive, and so overexpression of even wildtype protein gives phenotypes that may not be related to the disease phenotype arising from mutation. This is the case for mutation in *TARDBP* encoding the TDP-43 protein, *FUS* encoding the fused in sarcoma protein, and several other ‘ALS proteins’ [reviewed in (De Giorgio et al. 2019)].

**Table 1:** Genetically humanised neurodegeneration mouse models referred to in the text

	Disease	Model (gene)	Referred to in
Transgenics	AD/tauopathy	5xFAD (*APP, PSEN1*)	Choi et al. (2018), Devi et al. (2010), Neuner et al. (2019)
		APPPS1 (*APP, PSEN1*)	Ali et al. (2018), Bacioglu et al. (2016), Espuny-Camacho et al. (2017), Lesuis et al. (2018), Liao et al. (2018), McGinley et al. (2018), Wang et al. (2003)
		APPSw-NSE (*APP*)	Lee et al. (2015)
		3xTgAD (*APP, PSEN1, MAPT*)	Ager et al. (2015), Blurton-Jones et al. (2009), Halagappa et al. (2007), Hirata-Fukae et al. (2008))
		hTau (*MAPT*)	Gratuze et al. (2017)
		J20 (*APP*)	Lee et al. (2018)
		Tg2576 (*APP*)	Callahan et al. (2001), Dong et al. (2004), Farr et al. (2014), Kim et al. (2015), Lee et al. (2002), Schafer et al. (2015)
		P301S-Tau (*MAPT*)	Bacioglu et al. (2016), DeVos et al. (2017))
		ht-PAC-E10 + 14 (*MAPT*)	Sud et al. (2014))
	HD	R6/2 (*HTT*)	Hockly et al. (2002)
		R6/1 (*HTT*)	Harrison et al. (2013), Pang et al. (2006))
		YAC128 (*HTT*)	Ehrnhoefer et al. (2018), Moreno et al. (2016)
		C6R (*HTT*)	Ehrnhoefer et al. (2018)
		N171–82Q (*HTT*)	Corrochano et al. (2018), Potter et al. (2010)
		BACHD (*HTT*)	Kordasiewicz et al. (2012), Monteys et al. (2017)
	ALS	SOD1-G93A (*SOD1*)	Acevedo-Arozena et al. (2011), Ari et al. (2014), Bilsland et al. (2010), Deitch et al. (2014), Dupuis et al. (2004), Gaj et al. (2017), Garbugino et al. (2018), Goutman et al. (2015), Gurney et al. (1994), Hamadeh et al. (2005), Heiman-Patterson et al. (2005), (2011), Kang et al. (2013), McCampbell et al. (2018), Olivan et al. (2014), Pfohl et al. (2015), Riar et al. (2017), Williams et al. (2009a), Zhao et al. (2006)
		SOD1-L126delTT	Watanabe et al. (2005)
		C9-BAC (*C9orf72*)	Liu et al. (2016)
		Prp-TDP-43-A315T (*TARDBP*)	Coughlan et al. (2016)
		TDP-43-A315T, -G348C (TARDBP)	Pozzi et al. (2019)
		hgFUS-R521C, -R521H (*FUS*)	Lopez-Erauskin et al. (2018)
	PD	A53T-αS, A30P-αS (*SNCA*)	Bacioglu et al. (2016)
	SMA	hSMN2 (*SMN2*)	Hua et al. (2011), Passini et al. (2011), Porensky et al. (2012), Williams et al. (2009b)
Targeted to non-endogenous locus	ALS	Tau-ON-hFUS-P525L (*FUS*)	Sharma et al. (2016)
		TDP-43-M337V-ROSA26 (*TARDBP*)	Gordon et al. (2019), Sleigh et al. (2018)
Targeted to endogenous locus	AD	APP-NL, -NL-F, -NL-G-F (*APP*)	Saito et al. (2014)
		hAPOE2, -E3, -E4 (*APOE*)	Huynh et al. (2017), Knouff et al. (1999), Liao et al. (2018), Shi et al. (2017), Sullivan et al. (1997), (1998)
	HD	CAG140 KI (*HTT*)	Stefanko et al. (2017)
		HDH(CAG)150 (*HTT*)	Heng et al. (2007)
	ALS	FUS-Delta14 (*FUS*)	Devoy et al. (2017), Sleigh et al. (2018)
Whole chromosome humanisation	DS	Tc1 (Chr. 21)	Deveson et al. (2018), O’Doherty et al. (2005), Wilson et al. (2008)

Genome engineering technologies allow genetic humanisation at predefined genomic sites to create humanised knock-in mice, made through targeting via homologous recombination in mouse embryonic stem cells or directly in zygotes via CRSIPR/Cas9 genome-editing technologies. One knock-in humanisation approach is the insertion of human sequences into non-endogenous ‘safe harbour’ loci—so named because targeted insertion at these loci generally does not affect the viability or fertility of the mouse. *Rosa26* is one commonly used ‘safe harbour’ locus, which has been used to produce a number of neurodegeneration-relevant transgenic lines, for example, TDP-43-M337V (ROSA26) mice, which express a pathological *TARDBP* human gene under the control of the human *TARDBP* promoter (Gordon et al. 2019). While expressed at relatively low levels compared to transgenic models, the precise impact of expression at *Rosa26* on cellular and temporal expression is unclear.

‘Physiological’ models are those that maintain gene-of-interest expression at endogenous levels (in the appropriate cellular and temporal context). These include mutants generated by random N-ethyl-N-nitrosourea mutagenesis of the mouse genome, for example, to produce the Sod1-D83G mouse, which has an identical mutation (aspartic acid mutated to glycine at residue 83 ‘D83G’ of superoxide dismutase 1) as found in human SOD1-ALS families (Joyce et al. 2015). Alternatively, ‘physiological’ models can be engineered by the targeted knock-in endogenous locus, via homologous recombination in mouse embryonic stem cells or directly in zygotes via CRISPR/Cas9 genome editing. At the smallest scale, targeted knock-in has been used to introduce point mutations associated with human TDP43-ALS into the endogenous mouse *TARDBP* gene (Fratta et al. 2018; White et al. 2018). Physiological humanised models are now also being generated via targeted replacement of mouse sequences by orthologous human sequences (Zhu et al. 2019). Such physiological humanised knock-in models include those for studying AD, whereby finescale humanisation of only a handful of mouse amino acids in the amyloid precursor protein (APP) encoded by the mouse *App* gene, resulted in a fully human amyloid beta peptide; and recreating unique human biochemistry, such that these mice provide an excellent physiological model of human amyloid beta protein deposition (Saito et al. 2014). Going further, whole genes (Leidy-Davis et al. 2018; Wallace et al. 2007) and entire gene clusters (Lee et al. 2014; Macdonald et al. 2014) can now be humanised by genome engineering. At the largest scale, genetic humanisation can be achieved by integration of entire human (or human artificial) chromosomes via microcell-mediated chromosome transfer, a technique which has been used to model human trisomy 21 that gives rise to Down syndrome (O’Doherty et al. 2005).

We note that non-coding sequences, including the promotors, may be different between mouse and human resulting in species-specific differences in gene expression that in some cases will affect phenotypes (Deveson et al. 2018). Genomically humanised mouse models that include promotors can start to tease out these effects (Wilson et al. 2008).

#### Cellular humanisation: generating human: mouse chimeras at the tissue level

The advent of human iPSCs has enabled patient-specific disease-in-a-dish modelling, but 2D or even 3D culture systems cannot recapitulate the complexity of the CNS in vivo. Furthermore, the differentiation of human iPSCs into mature CNS cell types beyond an embryonic-like state (and capable of developing age-related neurodegenerative phenotypes) has proved to be challenging (Vera and Studer 2015). However, studies of chimeras show a variety of human iPSC-derived neural or glia precursors survive, differentiate into diverse lineages, mature, and functionally integrate when engrafted into the mouse brain or spinal cord (Chen et al. 2015, 2016; Espuny-Camacho et al. 2013; Goldman et al. 2015; Krencik et al. 2011; Wang et al. 2013). These chimeric humanised cellular models can be used to study cell intrinsic pathogenic mechanisms of neurodegeneration within patient iPSC-derived human CNS cells in vivo in the context of the amazingly complex mouse brain.

Patient-derived cells used may not necessarily be neurons. In human:mouse glia chimeras, the engrafted human glia precursors outcompete their mouse counterparts, such that over time the chimeric mice harbour an astroglial network dominated by human astrocytes. Models of hypomyelination can harbour an almost fully humanised oligodendrocyte network (Goldman et al. 2015; Krencik et al. 2011; Wang et al. 2013; Windrem et al. 2014). Furthermore, human astrocytes are distinct with respect to their size, complexity, and diversity; attributes that can be recapitulated in human:mouse glia chimeras in vivo and may be significant for human neurodegenerative processes (Chen et al. 2015; Han et al. 2013; Krencik et al. 2011; Oberheim et al. 2009).

## Four components of phenotype

Is there a role for the standardised, usually inbred, laboratory mouse to help us reach strategic decisions about human healthcare for subpopulations in real life? We believe humanised mouse models are already doing so, and must be a part of precision medicine networks in the future.

Here, we consider how mouse models of neurodegeneration contribute to precision medicine, looking at four major factors that can affect phenotype: genetics (including monogenic causative mutations, epigenetics, and the effect of genetic background), environment, ageing, and stochasticity. We then consider the contribution of these models to therapies.

### Genetics

#### Mouse models pave the way for understanding pathomechanism related to individual mutations

Genetically mutant mice have been used for decades to model and drill into fundamental pathogenic mechanisms for a wide spectrum of human monogenic neurodegenerative disorders, and so we do not discuss them in detail here.

Some neurodegenerative disorders, such as HD or other polyglutamine disorders, are defined both by their clinical presentation and a singular genetic alteration. Other diseases including ALS/FTD, AD, and PD are defined principally by their clinical presentation, and have heterogeneous causative mutations. For example, > 25 genes can cause dominantly inherited monogenic familial ALS and/or FTD, although we do not yet understand the genetic changes conferring susceptibility in most sporadic ALS cases (90% of patients). Humanised genetic mouse models have provided vital insights into the pathomechanisms underlying ALS, showing that mutations in different genes cause distinct pathological changes—from toxic protein accumulation (Liu et al. 2016) to cytoplasmic mislocalisation of RNA-binding proteins (Devoy et al. 2017; Gordon et al. 2019; Sharma et al. 2016) to axon transport deficits (Bilsland et al. 2010; Sleigh et al. 2018) to defective protein synthesis (Lopez-Erauskin et al. 2018) to the involvement of oligodendrocytes (Kang et al. 2013), and many other alterations (Taylor et al. 2016)—that may lead to motor neuron degeneration and/or FTD.

Humanised mouse models can be used to model individual patient mutations, including rare mutations, such as the SOD1-L126delTT mutation found in a single Japanese family with distinctive ALS pathology among SOD1 mutation carriers (Watanabe et al. 2005), and the aggressive FUS-Delta14 mutation from a single sporadic early-onset ALS patient modelled in mice through a partial humanisation knock-in strategy (Devoy et al. 2017). These diverse models will give us a better understanding of the function of different genes implicated in neurodegenerative diseases, and how different mutations in the same gene can impact on disease pathology and the potential implications for treatment. Modelling rare variants with humanised sequence will be particularly useful in developing therapies that target specific human mutations, mutant transcripts, or proteins.

These mechanistic studies provide invaluable information to help in predicting which patients may be responsive to specific therapies. Historically, in clinical drug trials for neurodegenerative disorders, treatments have not stratified patients by the underlying genetic causes, although the landscape is changing and humanised mouse studies are guiding more precise approaches. For example, arimoclomol is a drug that induces the heat shock protein response and was shown to be effective at ameliorating neuromuscular pathology and function in SOD1-G93A mice, and is now in clinical trials for SOD1-ALS patients (Benatar et al. 2018; Kieran et al. 2004). This approach may be essential for achieving significant treatment efficacy, in particular for treatments that target underlying mechanistic causes, which are diverse and likely linked to specific genetic factors.

#### The consequences of genetic variation

Humans are considerably more variable at the genetic level than inbred laboratory mice and this affects clinical outcome at all levels and thus decisions about precision medicine. Different alleles of a single modifier gene may radically affect our susceptibility and response to disease. For example, the *APOE* locus (encoding Apolipoprotein E which functions in lipoprotein metabolism) has three variants (differing at 2 residues) in the human population (*APOE2*, -*E3*, and -*E4*), each of which has dramatic effects on risk for both late-onset AD (-*E4* confers risk) and cardiovascular disease (-*E4* also confers risk, as does the rare-*E2/*-*E2* haplotype that also confers risk for hyperlipoproteinemia). These risks are imparted through varying affinities for lipoproteins and lipoprotein receptor-binding capabilities: both the -*E2* allele and common-*E3* allele preferentially bind to small phospholipid-enriched high-density lipoproteins, although APOE2 is defective in binding to lipoprotein receptors, while APOE4 distinctly binds larger triglyceride-enriched very low-density lipoproteins (Mahley 2016). Mice only have the ancestral -*E4* variant, although evolutionary divergence between mouse and human results in this allele behaving similarly to the lower risk human -*E3* allele (i.e. affinity for high-density lipoproteins). Targeted genomic humanisation via knock-in to replace the endogenous mouse locus with each of the three human variants resulted in the humanised variants binding lipoproteins as they do in humans (Knouff et al. 1999; Sullivan et al. 1997, 1998), providing a unique toolkit to understand the distinct impact of these alleles on disease. With respect to neurodegeneration, humanised APOE mice have uncovered pathomechanisms specific to -*E4* carriers, showing that the presence of APOE4 aggravates tau-mediated neurodegeneration and neuroinflammation (Shi et al. 2017).

Genetic background, whether mouse or human, is critical for modulating disease. Modifier genes, defined as genes that are not disease causative, but can modulate disease outcomes ranging from susceptibility to onset to disease progression, play a major role in pathogenesis. However, we largely study mice on a single inbred genetic background, akin to studying disease in a single patient. Some studies have looked at the effects of different inbred genetic backgrounds on mouse models of disease, for example, as in SOD1-G93A ALS mice, demonstrating strain-specific effects on disease onset, progression, and lifespan (Acevedo-Arozena et al. 2011; Heiman-Patterson et al. 2011). A recent study used the BXD genetic reference panel of recombinant inbred strains—derived from C57BL/6 and DBA/2 lines segregating for > 4.8 million SNPs—to understand how background genetic variation can impact AD, using crosses to humanised 5xFAD transgenic mice (a model of amyloid deposition) (Neuner et al. 2019). Genetic background modified the expressivity of known late-onset AD-risk alleles, and modified the expressivity of cognitive phenotypes, similar to the variation seen in human AD. This included a DBA/2 parental SNP in *APOE* associated with poorer performance in contextual fear acquisition. Furthermore, transcriptomic changes in aged AD-BXD mice showed higher concordance with transcriptomic profiles from late-onset AD patient data sets, compared to inbred AD models. Thus, humanised mouse models incorporating genetic variability can improve the modelling of complex human diseases and provide a valuable tool to understand genetic modifiers of disease, and how these impact on treatment strategies for individual patients.

Cellular humanisation offers a unique perspective into understanding how human cells, from any given genetic background, succumb to neurodegeneration. Recently, the fate of GFP expressing human PSC-derived cortical neuron precursors engrafted into the brains of neonatal transgenic AD mice (APPPS1) was studied in relation to resident host neurons (Espuny-Camacho et al. 2017). APPPS1 mice display early-onset amyloid pathology, but minimal neuron loss (Radde et al. 2006); in contrast, human PSC-derived engrafted neurons, exposed to host amyloid beta plaques, underwent significant neuronal death by 6 months of age accompanied by signs of neuroinflammation and neurotic dystrophy (Espuny-Camacho et al. 2017). As controls, no neuron loss was observed in mouse neurons engrafted into APPPS1 mice, nor in human neurons engrafted into wildtype mice. Thus, human cortical neurons are distinctly vulnerable to non-cell autonomous neurodegeneration processes associated with amyloid beta plaque exposure compared to mouse neurons. This study highlights that transplanting healthy cells into host-patient brains (i.e. for cell replacement therapy) with an advance diseased state may be ineffective for AD, as disease pathology could be transmitted from cell to cell.

This model system, combined with patient-derived iPSCs, offers the opportunity to analyse AD neurodegeneration within individuals or groups with distinct genetic backgrounds. Indeed, the authors demonstrated enhanced neurodegeneration of iPSC-derived human neurons from a patient with an FTD-causing *MAPT* mutation, exceeding the neuron loss observed in healthy human iPSC-derived neuron chimeras (Espuny-Camacho et al. 2017), thus providing insight into human-specific cell-autonomous pathomechanisms.

Human:mouse glia chimeras using patient iPSC-derived glia precursors engrafted into healthy donor mice have been successfully used to model ALS, DS, and HD in the genomic and cell-autonomous context of individual patients’ astroglia (Benraiss et al. 2016; Chen et al. 2014, 2015; Osipovitch et al. 2019; Qian et al. 2017). Similarly, generation of human:mouse neuronal chimeras (Chen et al. 2016; Espuny-Camacho et al. 2013) using patient iPSC-derived neuronal precursors has been used to model DS neurodegenerative processes within the context of individual patient cortical neuron dynamics (Real et al. 2018). The next step is to model these processes with genetically divergent human iPSCs, into genetically divergent host mice, to see the full spectrum of biological complexity that gives rise to neurodegeneration.

#### Risk associated with sex

Another genetic modulator of disease phenotype is sex, which alters risk for many neurodegenerative disorders, including AD (increased female risk) and PD and ALS (both with increased male risk). The reasons underlying these differential risks are not understood, but could be related to steroid sex hormone differences during development and/or during adult life impacting on brain morphology and function (Qiu et al. 2018), metabolism (Zhao et al. 2016), or cellular processes such as autophagy (Congdon 2018). Studies in humanised transgenic mouse models of AD (examples include Tg2576, 3xTgAD, APPPS1, and 5xFAD models) have underpinned the differential gender risk by uncovering differential development of pathological changes, including an earlier onset of amyloid beta deposition, increased amyloid beta production, and more aggressive amyloid beta pathology in female model mice (Callahan et al. 2001; Hirata-Fukae et al. 2008; Wang et al. 2003). Humanised mouse models have been used to dig deeper into mechanism; for example, pathological differences have been attributed to female-specific age-related increases in synaptic zinc, thought to contribute towards the aggregation of amyloid beta (Lee et al. 2002). Additionally, stress, which as discussed below acts as an environmental risk factor for AD, has been shown to drive marked female-specific increases in amyloid pathology in the hippocampus, associated with increased levels of in APP and BACE1 (cleaves APP to produce amyloid beta), in an AD mouse model (Devi et al. 2010). Overall, these marked pathological and mechanistic differences between the sexes suggest that AD treatments may need to be tailored to gender; backed up by a study in AD mice showing that calorie restriction ameliorated pathology only in females (Schafer et al. 2015).

In the widely used SOD1-G93A humanised transgenic ALS model, female mice exhibit later onset of disease and prolonged survival versus males in some but not all inbred strains, demonstrating genetic background-dependent effects, consistent with an increased incidence of ALS in males (Heiman-Patterson et al. 2005; Pfohl et al. 2015). Female-specific protection was subsequently linked to the estrogen-receptor alpha-mediated mitochondrial unfolded protein response (Riar et al. 2017).

Ultimately, studies in both sexes in humanised mouse models of neurodegeneration will serve to understand vulnerabilities or protective mechanisms associated with sex that can aid in the development of new treatments (Turner 2001). These and human studies also highlight the need to stratify clinical trials by sex to reflect sex differences in disease pathology and mechanism, and to more accurately assess the efficacy of treatments.

### Environment

Even in the (fairly rare) neurodegenerative diseases caused by a highly penetrant monogenic mutation, environmental factors have a role in determining outcomes (penetrance, severity, onset of disease). For example, phenotype in monogenic humanised genetic mouse models of HD (R6/2) can be improved by environmental enrichment (such as introducing toys, ladders, varied bedding), in ways we need to better understand, to give best advice about lifestyle while treatments are being developed in humans (Hockly et al. 2002). The long-term effects of almost everything we consider important for human disease manifestation, including early life stress, can be tested in mice—with the rather large proviso that mice have their own biology, including species-specific behaviours, and are not simply small humans.

One key area in which humanised mice can tell us about the effects on environment lies in the study of weight, metabolism, and diet. Weight is an important trait in all human neurodegenerative disorders. Alterations in weight reflect changes in whole-body metabolism induced by disease (Kitamura et al. 2017; Mariosa et al. 2017; Nordestgaard et al. 2017; Singh-Manoux et al. 2018) and body weight alterations (i.e. weight loss or weight gain) are common comorbidities found in neurodegenerative disease (Djousse et al. 2002; Emmerzaal et al. 2015; Hu et al. 2006; van der Burg et al. 2017; Wills et al. 2016). It is therefore not surprising that obesity and type II diabetes are risk factors for AD (Singh-Manoux et al. 2018). Conversely, higher Body Mass Index at onset and reduced weight loss through disease progression are beneficial in ALS (Peter et al. 2017). The weight of an individual is also a complex trait, pre-determined by genetics as well as environmental interaction (such as diet) and time-associated changes (ageing).

The study of external environmental contribution to weight and metabolic balance, through controlled diet, is an important entire field of research (Pistollato et al. 2018), not only in neurodegenerative diseases, but in other complex disorders, including diabetes, cardiovascular disease, and cancer. For precision medicine, it is being championed by the emerging field of Nutrigenomics, which focuses on the interaction of diet and genetics, and how specific genetic profiles can help determine the nutritional needs of individuals, in health and disease.

Many studies on nutrition and age-related disease, including many forms of neurodegeneration, incorporate dietary caloric restriction, as this appears to extend lifespan by stimulating autophagy via expression of Sirtuin 1 (Morselli et al. 2010). Dietary restriction, or related interventions such as intermittent fasting, have proven effective at ameliorating phenotypes in preclinical studies with genetically humanised HD (YAC128, C6R) and AD (3xTgAD) mouse models (Ehrnhoefer et al. 2018; Halagappa et al. 2007; Moreno et al. 2016). However, caloric or dietary restriction is not beneficial in every context, and mouse models are of enormous value to understanding how such interventions may be beneficial in the human population. For example, caloric restriction exacerbated tau aggregation in the brain of an obese humanised tau mouse model (htau) (Gratuze et al. 2017), and dietary restriction is detrimental in the SOD1-G93A ALS mouse model (Hamadeh et al. 2005). Accordingly, a ketogenic or high-energy diet is protective in SOD1-G93A mice (Ari et al. 2014; Dupuis et al. 2004; Olivan et al. 2014; Zhao et al. 2006), and similarly for humanised Prp-TDP-43-A315T transgenic mice (Coughlan et al. 2016). Aside from modulating foods associated with energy metabolism, dietary supplementation with antioxidants has also been shown to be beneficial in humanised AD (APPPS1) mice (Ali et al. 2018).

Tied in with metabolism, weight, and diet is exercise, which may prove an important consideration for precision medicine strategies for many neurodegenerative diseases. Exercise is considered beneficial by improving cardiovascular health and strength, and boosting metabolism through effects on skeletal muscle (Booth et al. 2015), but has other benefits associated with neurological function, including improving mental health and cognition. Physical inactivity is a major risk factor for AD (Norton et al. 2014), and the benefits of exercise for AD have been extensively studied in genetically humanised mice (Shepherd et al. 2018). For example, in humanised 5xFAD AD animals, exercise induced cognitive benefit through increasing adult hippocampal neurogenesis and elevating BDNF levels, so providing a novel AD therapeutic avenue (Choi et al. 2018). Similarly, in humanised mouse models of HD (R6/1 and CAG140 KI mice), exercise primarily improving cognitive functions (Harrison et al. 2013; Pang et al. 2006; Stefanko et al. 2017). However, the form of exercise could be critical: for example, prolonged strenuous exercise was detrimental for motor-function and lifespan in humanised HD (N171–82Q) mice (Corrochano et al. 2018; Potter et al. 2010); perhaps not surprisingly given that HD is a neuromuscular wasting disorder. Similarly for ALS, another neuromuscular wasting disorder, negative effects of exercise have been shown in SOD1-G93A ALS mice (Garbugino et al. 2018), consistent with a recent patient study that found a positive correlation between physical activity and risk for developing ALS (Visser et al. 2018).

Finally, stress is also a crucial factor modulating the onset and progression of neurodegenerative disease, as well as many other pathologies. It has been demonstrated that exposure of AD humanised mouse models to different kinds of stressors exacerbates both intracellular and extracellular pathologies associated with AD (reviewed in (Justice 2018)). For example, isolation stress increased amyloid beta deposition and perturbed memory in AD (Tg2576) mice (Dong et al. 2004). Similarly, in another AD (APPPS1) mouse model, early life stressors increased amyloid pathology and impaired cognitive performance in 12-month old mice, responses that could be prevented by transient blocking of glucocorticoid receptors, providing a potential therapeutic intervention targeting glucocorticoid hormonal increases that occur in response to stress (Lesuis et al. 2018). While stress is principally environmental, individual responses to physical and mental stressors partly depend on patient-specific genetic and epigenetic traits, which should also be considered from a precision medicine standpoint.

### Ageing

Ageing is the major risk factor for almost every neurodegenerative disorder. The exact correspondence of human and mouse ages may be debated, but for most strains, 3 months of age is a young adult and a mouse over 18 months of age is considered ‘old’. While there are clearly huge differences between human and mouse in rates of ageing and of lifespans, we share the effects of senescence, defined by ‘biomarkers’ such as loss of pigment in the hair and weight gain. Thus, ageing mice in health and disease provide a model system for studying ageing, including effects on neurodegeneration. As with all phenotypes, lifespan and rate of ageing depend on the genetic background of the mouse, with a long-lived strain such as C57BL/10 living for an average of 826 days for males and 693 days for females, whereas BALB/c live on average 539 for males and 575 for females (Festing 1979). Indeed, the SAM inbred strains were developed to be either senescence-prone (SAMP) or senescence-resistant (SAMR), specifically for studies of age-related phenotypes (Takeda 2009; Takeda et al. 1981), and these mice have provided a model to study the transition from healthy ageing to neurodegeneration, notably in AD (Diaz-Moreno et al. 2018).

For modelling neurodegenerative disease, mice need to have a relevant mutation, and they need to be studied at a range of ages. Overexpression mutants with human transgenes are the still the most widely used models of neurodegeneration but they may have a severe disease, sometimes with a relatively short lifespan that can make study of ageing-related processes difficult. As discussed above, ‘low-copy’ transgenics can exhibit a slower disease course, but gene dosage is often still artificially high; for example, ‘low-copy’ SOD1-G93A mice have between 4 and 10 copies of human *SOD1* (Acevedo-Arozena et al. 2011; Deitch et al. 2014). However, the use of knock-in models that have a slower rate of progression, with physiological expression of the mutation, is more informative for both early-stage disease processes and for following the effects of ageing; such models include genetically humanised FUS-ALS (Devoy et al. 2017) and HD (Heng et al. 2007) knock-in mice that exhibit age-dependent, progressive neurodegeneration.

A key issue for precision medicine and modelling human disease is how age affects the efficacy of treatments. For example, the effect of physical exercise has been debated in ALS and in AD as above. Studies in mouse and human show that physical exercise may reduce risk of AD, but a recent large patient study showed exercise had no positive effect (and may have negative effects) (Shepherd et al. 2018). Thus, while lifelong exercise may have beneficial effects with regard to AD risk, exercise in older age in patients should be viewed independently. In the context of precision medicine, it is possible that particular exercise regimes may be beneficial in some patients, but we need considerably more studies of age effects, genetics, exercise regime, etc. to draw conclusions. However, this area is vitally important, because exercise is a relatively straight-forward and cheap treatment that does not require patient hospitalisation.

### Stochastic factors

A recent study showed that random somatic mutation in the brain contributes to development of sporadic AD, through the creation of multiple reverse-transcribed copies of *APP* reinserted into the genomes of individual neurons, as discovered in human brain samples and a humanised AD mouse, (J20 transgenic animals, which overexpresses human APP with 2 AD mutations) (Lee et al. 2018). Thus, stochastic events (closely tied to ageing) may have a profound disease causative effect in neurodegenerative disease, as is the case in cancer. How we model this in the mouse nervous system, to corroborate cause and effect and to further explore mechanism, is to be determined, but lessons can be learned from cancer research, where researchers have engineered sophisticated mouse models that produce somatic mutations, to pave the way for therapies for human disease (Fisher et al. 2009; Maresch et al. 2016; Sanchez-Rivera et al. 2014).

Precision medicine experts (in computational biology and other fields) can help model and understand how stochastic events contribute to disease processes in human and in mouse. This is essential for our understanding of mechanism: for example, human data on ALS have shown that at least some forms may require six genetic lesions or ‘hits’ through life, to result in this neurodegenerative disease (Al-Chalabi et al. 2014).

## Humanised mouse models for therapeutic strategies in precision medicine

Gene therapy is a promising new approach for the treatment of neurodegenerative disease. Therapies utilising antisense oligonucleotides (ASOs), a method of gene silencing, have the potential to ameliorate a range of neurodegenerative disorders. ASO-based strategies rely on binding to complementary RNA, hence humanised genetic models of neurodegeneration, which carry human coding sequences of interest, are an ideal system for preclinical testing. For example, ASOs, administered typically via intraventricular or intrathecal injection, have proven effective at silencing pathogenic gene products in genetically humanised mouse models of ALS (McCampbell et al. 2018), HD (Kordasiewicz et al. 2012), AD (Farr et al. 2014), and tauopathy (DeVos et al. 2017; Sud et al. 2014). Genetically humanised mice have also been used to assess the impact of a known modifier gene for AD, *APOE4*: ASO administration to double-humanised APOE (knock-in)/APPPS1 (transgenic) AD mice resulted in successfully reducing amyloid beta plaque pathology in both -*E4/E4* (risk associated) and -*E3/E3* (non-risk associated) mice, but only when applied before the onset of amyloid deposition, offering a valuable perspective on the utility of this treatment in the general population, and the need to carefully time treatments in human patients (Huynh et al. 2017). Notably, an ASO approach tested extensively in genetically humanised transgenic *SMN2* mice (a gene not present in mouse) blocks an intronic splicing silencer in human *SMN2,* which increases full-length *SMN2* isoform expression and compensates for loss of *SMN1* that causes SMA (Hua et al. 2011; Passini et al. 2011; Porensky et al. 2012; Williams et al. 2009b). These pioneering mouse studies have been used as proof of principle to drive many of these therapies into clinical trials, and in the case of *SMN2* ASO therapy, approval for clinical use (Schoch and Miller 2017).

The emergence of CRISPR/Cas9 technology is providing an opportunity to correct or nullify pathogenic genes permanently at the genomic level. Similar to the above-mentioned mRNA-based targeting strategies, genetically humanised mice offer the perfect model system for preclinical testing of gene-editing approaches that can be directly translated to the clinic. Recently, an AAV-based delivery of CRISPR/Cas9 components has been proven to work effectively to reduce hSOD1 levels in the SOD1-G93A humanised mouse model, delaying disease onset and extending survival (Gaj et al. 2017), again providing proof of principle for clinical trials.

A third major therapeutic use of humanised mice is their use in testing immunotherapies that target human proteins. For example, AAV delivery of an human anti-TDP-43 antibody was recently shown to ameliorate pathology in humanised *TARDBP* transgenic (TDP-43-A315T, -G348C) mouse models (Pozzi et al. 2019). Similarly, for AD, an antibody specifically targeting aggregated forms of the AD-risk variant APOE4 was administered to APOE4/APPPS1 double-humanised mice and shown to reduce amyloid beta deposition, implicating APOE4 aggregation in the formation of amyloid plaques (Liao et al. 2018). However, antibodies directly targeting amyloid beta have been tested in humanised mice for over two decades, and while some have progressed through to clinical trials, none have made it to clinic (Kohyama and Matsumoto 2015; Panza et al. 2019). Part of the disconnect between bench and clinic could be due to genetic heterogeneity among AD patients in trials versus the few rare forms of familial AD modelled in mice, highlighting the need to better stratify and match patients to the types of mutations used in mice, at least in initial efficacy studies. Also, a more diverse set of animal models is warranted to better understand disease diversity in patients, and of course the timing of human disease manifestation and treatment may be different from testing cohorts of genetically similar mutant mice.

Cell replacement therapies (CRT) offer the potential to replace neurons lost to neurodegeneration, and thus retain or restore neurological function. Indeed, early CRT clinical trials to treat PD patients 3 decades ago showed promise, with human foetal ventral mesencephalon (hVM) engraftments demonstrating notable motor improvements in small cohorts of patients (Freed et al. 1992; Lindvall et al. 1990; Spencer et al. 1992; Widner et al. 1992), although in subsequent, larger trials patients failed to consistently respond positively, with some suffering adverse effects (Freed et al. 2001; Olanow et al. 2003). Such therapy is still not routine today, likely due at least in part to the genetic and phenotypic heterogeneity of patients involved, highlighting the importance for research into the application of precision medicine to fine-tune CRT strategies. The use of animal models has a crucial role to play, not least in the development of the technology itself. Prior to the aforementioned clinical trials, pioneering preclinical work engrafting hVM tissue into a rat chemically induced PD model demonstrated vital proof of principle (Brundin et al. 1986). In more recent years, human PSCs (embryo derived or induced) are showing great promise as a potentially infinite source of cells for CRT, versus the very limited supply of human foetal tissue.

This promise is borne out in cellular humanisation animal studies whereby human PSC-derived dopaminergic neurons have been grafted into the brains of mouse, rat, and non-human primate models of PD, with positive outcomes for ameliorating phenotypes (Grealish et al. 2014; Kikuchi et al. 2017; Kriks et al. 2011; Roy et al. 2006), although the PD models were all chemically induced, and it may be prudent to test this treatment strategy in genetic models of disease. Nevertheless, human trials are on the horizon (Barker et al. 2017).

In AD, similar efforts have been made to develop CRT using foetal human neural tissues, adult human neural and non-neural tissues, or human PSC-derived neural progenitor cells (NPCs) engrafted into animal models (Fang et al. 2018). Most of the models used in these preclinical AD studies are transgenic mice bearing human familial AD-mutated gene copies, and predominantly mutated human APP (Ager et al. 2015; Blurton-Jones et al. 2009; Lee et al. 2015; McGinley et al. 2018). While this is encouraging, over 95% of AD cases are non-familial, caused by poorly understood interactions between multiple genetic variants (notably the 3 human *APOE* variant alleles) and environmental/lifestyle factors; thus, the response to treatment in these patients is less predictable. It is likely that many patients (with specific/distinctive AD pathogenic causative mechanisms) will not be responsive to a one-size-fits-all neural cell transplantation intervention. For example, astroglia make a significant contribution to AD pathogenesis, through dysregulation of inflammation that can influence amyloid beta levels, or through production of APOE4 variants (astrocytes primarily produce APOE4 and regulate neuronal APOE) (Gonzalez-Reyes et al. 2017; Harris et al. 2004; Oksanen et al. 2017). Thus, transplantation of neurons into an astrocyte-driven toxic environment may not be beneficial, whereas transplant of astrocytes or glial progenitor cells (GPCs) may be more appropriate (Goldman et al. 2015).

Furthermore, animal studies have already provided evidence that stratifying disease by the largest risk factor for AD, age, dramatically impacts on treatment success (Kim et al. 2015), demonstrating the potential for predicting success in patients considering other common risk factors, such as APOE4 carrier status, for which there are humanised mouse models readily available (Knouff et al. 1999; Liu et al. 2017; Shi et al. 2017).

Similar conclusions can be drawn for preclinical human xenograft studies for ALS, from which many studies have demonstrated improvements in transgenic mice and rats, paving the way for early clinical trials evaluating (and successfully demonstrating) safety (Goutman et al. 2015, 2018). Preclinical animal studies were conducted almost exclusively in SOD1-G93A transgenic rodents; however, *SOD1* mutations are only 2% of ALS cases (< 10% ALS cases are familial; ~ 20% of these have *SOD1* mutations). Again, distinct genetic causes of ALS likely need distinct evaluation for treatment efficacy. Several mouse models for ALS exist, representing all the major ALS causative genes (*C9orf72*, *SOD1*, *TARDBP*, *FUS*, etc.), including new knock-in models discussed in this review (De Giorgio et al. 2019), which can be used to customise distinct strategies for CRT in patients.

The most likely, and plentiful, source of cells for CRT are human iPSCs; however, patient-derived autologous grafts bear a significant caveat—disease-causing mutations will remain and thus may retain pathological changes. One route around this problem is to correct pathological mutations in iPSCs via gene editing. In many cases of dominant mutations, for example, the HD-causing Huntingtin allele, mutant-allele-specific editing is desirable to avoid targeting potentially essential non-pathogenic gene copies. This can be achieved through a precision medicine approach by CRISPR/Cas9 targeting SNP variants proximal to pathogenic alleles (Chao et al. 2018; Shin et al. 2016). Such an approach has been validated in humanised genetic mouse models of HD (Monteys et al. 2017). However, this is not currently possible for the majority of AD, PD, and ALS patients who are ‘sporadic’ cases, as they do not carry known monogenic disease mutations.

Ultimately, early intervention or indeed prevention is the ideal therapeutic scenario. Humanised mouse models are proving their worth in understanding early neurodegenerative disease mechanisms, but the clinic needs to identify when these mechanisms have taken hold to inform when to begin therapies for individual patients and to identify disease onset as early as possible—and this means biomarkers. Biomarkers can also be used for stratification purposes to better understand disease diversity and how this might apply to treatment efficacy. The discovery and application of biomarkers to neurodegenerative diseases could ultimately help overcome the so-called “Valley of Death” effect, i.e. the failure to overcome between basic science and clinical application (Beach 2017). Humanised mouse models of neurodegeneration provide a tool to study, in depth, the progression of neurodegenerative disease and to uncover hallmarks of presymptomatic, early, and late disease processes.

Indeed, humanised mouse models have made fundamental contributions in this area. For example, a study of microRNA expression changes in SOD1-G93A transgenic mice identified mir-206 upregulation as a reliable marker of disease onset in animals (Williams et al. 2009a), which has subsequently also been observed in ALS patients (Ricci et al. 2018). Additionally, three humanised mouse models representing AD, tauopathies, and α-synucleinopathies including PD were used to identify neurofilament light chain (NfL) as a marker of neurodegenerative disease progression (Bacioglu et al. 2016), which has recently been translated to patient studies demonstrating serum NfL as a presymptomatic marker of familial AD (Preische et al. 2019).

## Conclusion and speculation

We are all (humans and mice) the result of complex of genetic (including epigenetic) and environmental interactions that occur pre-conception through to end of life. Humans and mice each have their own distinct advantages for medical research and we can (and should continue to) combine our understanding of both organisms to drive forward our understanding of biology and pathology. Human studies give us vast datasets of sometimes non-rigorously defined phenotypes with considerable ethical, legal, and other issues of privacy. Mice give us equally vast datasets but usually in limited genetic backgrounds for limited tests. Utilising defined congenic strains are invaluable for precisely identifying interactions of different genetic components—including pathogenic mutations, genetic background, and sex—with environmental variables and the process of ageing, without the confounding variable of genetic diversity. Genetic and cellular humanised mice provide added physiological relevance and provide the best models for preclinical testing of therapeutics. If we can integrate the advantages of both human and mouse studies, this will greatly aid precision medicine for preventative and patient-based healthcare.

For example, for clinical studies, humanised (and other) mice have clearly shown the need to stratify by genotype. ALS is the best illustration of this, where it is now clear that what appears to be a single disease, has over 25 different monogenic forms, affecting many individual pathways. Therefore, treatments developed for the SOD1-G93A mouse cannot be presumed to work in patients with TARDBP-ALS, for example. Many familial forms of neurodegeneration are rare, and so in many cases we have very few patients in which to study pathomechanisms related to a given disease-associated gene (for example, FUS mutations account for only ~ 4% of familial ALS cases). Furthermore, individual mutations within a given gene (i.e. rarer still, and in some cases only present in a single individual) may yield distinct disease outcomes that may inform precision medicine strategies—in such cases, mouse models can provide the sample sizes to drill into pathomechanism with statistical clarity. Delineating phenotypes, pathological mechanisms, and biomarkers according to specific genetic mutations in mice will aid greatly in informing stratification of patient groups to maximise the success of treatments in clinical trials.

Equally, for precision medicine studies, there is much to be learnt from the mouse studies as Lloyd and colleagues point out (Lloyd et al. 2015): the kind of large-scale, well-networked, high-throughput analysis that is essential for precision medicine (Berlin et al. 2017), is relatively routine (at least on a smaller scale) for the mouse community. Thus, we can use this experience to contribute to discussions about data management, accessibility, standardisation of phenotyping, etc. This also includes identification and analysis of critical metadata. Perhaps, the only area where the mouse and in particular humanised mouse models cannot contribute is in the issue of privacy and informed consent.

Equally, human diversity clearly highlights how we limit our understanding of neurodegeneration by working on single inbred lines, and the new finding of novel somatic mutation in AD, for example (Lee et al. 2018), illustrates how we will likely need new paradigms for modelling human neurodegeneration.

Finally, in studying humanised mouse models of neurodegeneration, we need to choose our model carefully. Genetic mouse models allow us to look at a systems level on the often underappreciated pleiotropic effects of mutation, and we now have the tools to engineer refined humanised alleles expressing mutated proteins at physiological levels. In comparison, humanised cellular mouse models are tissue- and cell-type specific in their humanisation, but capture a snapshot of human genetic heterogeneity, while also capturing unique attributes of human cells in an in vivo setting—for example, the unique vulnerability of human neurons or the complexity of human astrocyte subpopulations and connections—that cannot easily be achieved in genetic mouse models or in human cells in vitro. Combining these systems can offer even more powerful models to study human neurodegeneration in vivo, in cell autonomous and non-cell-autonomous contexts (Espuny-Camacho et al. 2017), and also provides excellent preclinical models to test therapeutics such as CRT.

If precision medicine is based on reviewing all the evidence to choose the optimal treatment for patients, then mouse studies must be included in this evidence. And of course, this is not only for treatment, but also for both prevention and diagnosis.

### Box 1. neurodegenerative diseases referred to in the text

*Alzheimer's disease, AD* AD is the most common form of dementia, accounting for ~ 2/3 of people with dementia. 95% of those affected are over 65 years of age. Extracellular amyloid plaques and intracellular tau tangles are deposited in the brain leading to clinical outcomes that include loss of memory, apathy, and depression; progressing to an inability to express and communicate; physical symptoms can include immobility and seizures.

*Amyotrophic lateral sclerosis, ALS* ALS is a progressive neurological disorder characterised by loss of motor neurons in the brain, brainstem, and spinal cord, resulting in weakness, muscle wasting, and ultimately respiratory failure. Symptoms usually present mid-late life with a disease course of approximately 5 years. The majority of ALS cases are sporadic (90%) with only a small proportion being familial (10%). ALS is genetically heterogeneous with more than 25 causative genes identified.

*Corticobasal degeneration, CBD* CBD is a rare neurodegenerative disorder characterised clinically as corticobasal syndrome (CBS) and pathologically as CBD by the deposition of abnormal tau protein in the somatosensory, premotor, supplementary motor cortices, brainstem, and basal ganglia. CBS is a combination of cortical signs (e.g. apraxia, aphasia, frontal dementia, parietal lobe sensory signs) and basal ganglia signs (e.g. rigidity, akinesia, limb dystonia, postural instability). CBS symptoms largely overlap with other parkinsonian and dementing illnesses, such as PD, PSP, and AD, making it difficult to diagnose.

*Down syndrome, DS* DS is the most common chromosomal abnormality found in newborns, with an incidence of up to 1 in 750 live births, increasing with maternal age. DS is caused by trisomy of chromosome 21. Phenotypic features include several neurodevelopmental abnormalities and difficulties with motor skills, memory, and learning. DS patients are at a greatly increased risk of developing AD, principally due to an extra copy of *APP,* on chromosome 21. DS is also associated with many different phenotypic outcomes which vary greatly in penetrance, and in expressivity in the individual. These include cardiac defects, gastro-intestinal diseases, periodontal diseases, and vulnerability to auto-inflammatory diseases.

*Frontotemporal Dementia, FTD* FTD is a group of related dementias resulting in brain atrophy of the frontal and temporal lobes, typically associated with behavioural changes and/or language impairments, and constituting a major, strongly inherited form of early-onset dementia. Onset is typically in mid-life, earlier than AD, and has a strong genetic and pathological overlap with ALS.

*Huntington disease, HD* HD is an autosomal-dominant neurodegenerative disorder caused by a polyglutamine tract expansion in the *HTT* gene, characterised by progressive chorea, dystonia, and cognitive and psychiatric symptoms including dementia. There is progressive, selective neuronal cell loss and atrophy in the caudate and putamen, which can be seen radiographically. Age of onset is around 30 years with a disease course between 5 and 20 years after the first clinical signs and symptoms are observed.

*Parkinson’s disease, PD* PD affects approximately 1% of the population over the age of 50 and is genetically heterogeneous with several loci identified causing PD. PD is progressive with onset in mid-to-late adulthood characterised by resting tremor, slowness of movements, muscular rigidity, bradykinesia, and postural instability; there may also be psychiatric features. Pathological features and histological hallmarks of PD include the presence of Lewy bodies and α-synuclein deposition; in contrast, autosomal recessive juvenile Parkinson’s disease does not have Lewy body pathology.

*Pick’s disease, PiD* PiD is a neurodegenerative disorder and a form of frontotemporal lobar degeneration. PiD is clinically extremely difficult to differentiate from AD and clinical features also overlap with CBD and PSP. PiD can only be confirmed histopathologically by lobar or circumscribed atrophy, Pick cells, and tau-positive Pick bodies.

*Progressive supranuclear palsy, PSP* PSP is a rare neurological disorder resembling PD. PSP has a wide clinical spectrum including aberrant movement, posture, gait, speech, swallowing, eye movements, mood, behaviour, and cognition. Pathologically, PSP is characterised by tau neurofibrillary tangles primarily localised to subcortical regions neurons and glia, which differentiates it from AD.

*Spinal muscular atrophy, SMA* SMA is an autosomal recessive neuromuscular disease affecting 1 in 6000–10,000 live births, predominantly caused by loss of function of the *SMN1* gene. SMA results in degeneration of the anterior horn cells of the spinal cord, symmetrical muscle weakness, and atrophy. SMA is divided into 4 sub-groups depending on age of onset, phenotypic severity, and survival. Type I (“non-sitters”), type II (“sitters”), type III (“walkers”), and type IV (adult‐onset).
